# Exposure to Volatile Organic Compounds May Contribute to Atopic Dermatitis in Adults

**DOI:** 10.3390/biomedicines12071419

**Published:** 2024-06-26

**Authors:** Kuo-Tung Tang, Yu-Sin Chen, Mey-Fann Lee, Tzu-Ting Chen, Chien-Chen Lai, Chi-Chien Lin, Yi-Hsing Chen

**Affiliations:** 1 Division of Allergy, Immunology and Rheumatology, Taichung Veterans General Hospital, Taichung 407, Taiwan; 2 School of Medicine, National Yang Ming Chiao Tung University, Taipei 112, Taiwan; 3 Ph.D. Program in Translational Medicine, National Chung Hsing University, Taichung 402, Taiwan; 4 Graduate Institute of Biotechnology, National Chung Hsing University, Taichung 402, Taiwan; 5 Institute of Biomedical Science, The iEGG and Animal Biotechnology Center, National Chung-Hsing University, Taichung 402, Taiwan; 6 Department of Medical Research, Taichung Veterans General Hospital, Taichung 407, Taiwan; 7 Institute of Bioinformatics and Structural Biology and Department of Medical Science, National Tsing Hua University, Hsinchu 300, Taiwan; 8 Institute of Molecular Biology, National Chung Hsing University, Taichung 402, Taiwan; 9 Graduate Institute of Chinese Medical Science, China Medical University, Taichung 404, Taiwan; 10 Rong Hsing Research Center for Translational Medicine, National Chung Hsing University, Taichung 402, Taiwan; 11 Department of Medical Research, China Medical University Hospital, Taichung 404, Taiwan; 12 Department of Pharmacology, College of Medicine, Kaohsiung Medical University, Kaohsiung 807, Taiwan; 13 Department of Post-Baccalaureate Medicine, College of Medicine, National Chung Hsing University, Taichung 402, Taiwan

**Keywords:** 1,3-butadiene, air pollution, atopic dermatitis, toluene, volatile organic compounds

## Abstract

Background: Volatile organic compounds (VOC) are major indoor air pollutants. Previous studies reported an association between VOC exposure and allergic diseases. Here, we aimed to explore the relationship between VOC exposure and atopic dermatitis (AD) in adults. Methods: We prospectively enrolled 31 adult AD patients and 11 healthy subjects as controls. Urine metabolite levels of VOCs, including 1.3-butadiene, acrylamide, benzene, toluene, and xylene, were all determined with liquid chromatography–mass spectrometry. The relationship between AD and log-transformed urine levels of VOC metabolites were examined using a multivariate linear regression model adjusted for age and sex. We also treated mouse bone marrow-derived cells (BMMCs) with 1,3-butadiene and toluene and measured the release of β-hexosaminidase. Results: Our results demonstrated that creatinine-corrected urine levels of N-Acetyl-S- (3,4-dihydroxybutyl)-L-cysteine (DHBMA), N-Acetyl-S-(2-carbamoyl-2-hydroxyethyl)-L-cysteine (GAMA), and N-Acetyl-S-(benzyl)-L-cysteine (BMA) were all elevated in AD patients compared with controls. In a multivariate linear regression model, creatinine-corrected urine levels of BMA (a toluene metabolite) and DHBMA (a 1,3-butadiene metabolite) appeared elevated in AD patients, although statistical significance was not reached after correction for multiple comparisons. In addition, 1,3-butadiene and toluene could stimulate BMMCs to degranulate as much as compound 48/80. Conclusions: Some VOCs, such as 1,3-butadiene and toluene, might be associated with AD pathogenesis in adults.

## 1. Introduction

Volatile organic compounds (VOCss) are major indoor air pollutants [1]. These compounds originate from paints, cleaning agents, and furnishings and adversely affect human health. Previous studies have linked VOC exposure to allergic diseases, despite some inconsistent findings [1]. Atopic dermatitis (AD) is a chronic relapsing dermatitis related to atopic diathesis [2]. Several studies have reported associations between ambient VOC concentrations and AD in children [3,4]. In recent decades, a significant disease burden of AD was uncovered in adults, with an even greater disease severity [5,6]. Nevertheless, no evidence has yet been presented regarding VOC exposure and adult AD.

The measurement of urine metabolites is a non-invasive method to determine internal doses after environmental exposure in humans. We hypothesized here that VOC exposure is associated with AD in adults. We then analyzed adult AD patients’ VOC exposure in terms of their metabolite concentrations in urine and determined the relationship between VOC exposure and AD.

## 2. Materials and Methods

### 2.1. Patients 

We prospectively enrolled 31 consecutive outpatients (≥20 years) with AD. AD was diagnosed based on the 1980 Hanifin and Rajka criteria [7]. Eleven healthy controls (HCs) without allergic diseases (allergic rhinitis, asthma, and atopic dermatitis) were recruited and they had a negative skin prick test to major allergens, as follows: Der p, Der f, and extracts of American cockroach [8], a biting midge *Forcipomyia taiwana* [9], *Cladosporium oxyspourm*, *Cladosporium cladosporioides*, and *Penicillium brevicompactum*. Our study protocol was approved by the Institutional Review Board of Taichung Veterans General Hospital (IRB TCVGH NO: CE16097A and CG19280B) and the written consent of all participants was obtained in line with the Declaration of Helsinki.

### 2.2. Determination of Clinical Parameters

Clinical parameters of AD patients were recorded as follows. Serum total IgE reports, which were measured with fluorescent enzyme immunoassay (ImmunoCAP-FEIA, Phadia, Freiburg, Germany), were collected in 23 patients within 3 months. Their analytical range is 2–5000 IU/mL and the normal range is <100 IU/mL. In case the derived total IgE values fell outside the analytical range, they were represented by the upper- or lower-bound value. Disease severity of AD was represented by the SCORAD (SCORing AD) index [10].

### 2.3. Determination of Urine VOC Metabolites

For each subject, we obtained first-voided morning urine (10 mL) which was then analyzed for a total of 6 VOC metabolites: 1,3-butadiene metabolite, N-Acetyl-S-(3,4-dihydroxybutyl)-L-cysteine (DHBMA); acrylamide metabolites, N-Acetyl-S-(2-carbamoyl-2-hydroxyethyl)-L-cysteine (GAMA) and N-Acetyl-S-(2-carbamoylethyl)-L-cysteine (AAMA); benzene metabolite, N-Acetyl-S-(phenyl)-L-cysteine (PMA); toluene metabolite, N-Acetyl-S-(benzyl)-L-cysteine (BMA); and xylene metabolite, N-Acetyl-S-(2,4-dimethylphenyl)-L-cysteine (2,4-DPMA). Quantification of these metabolites was performed using the API 4000 LC-MS/MS System (SCIEX, Concord, ON, Canada) along with the Agilent^®^ 1200 HPLC pumps (Agilent Technologies, Inc., Santa Clara, CA, USA). The limit of detection (LOD) was defined as three times the standard deviation of blank samples (Supplementary Table S1). All samples had no detectable urine levels of 2,4-DPMA and PMA and these metabolites were not analyzed. One sample GAMA concentration was below the LOD, and it was designated as half the LOD value. The dilutional effect of urine was adjusted for using the urine creatinine level, which was measured by the Jaffé method (Advia 1800; Siemens, New York, NY, USA). Creatinine-corrected VOC metabolite concentrations were used in the final analyses.

### 2.4. Isolation of Bone Marrow-Derived Mast Cells (BMMCs)

Bone marrow cells were obtained from the bone marrow of C57BL/6 mice (aged 4–6 weeks) and grown in RPMI1640 medium (Gibco, Thermo Fisher Scientific, Waltham, MA, USA) containing 30% WEHI-3B-conditioned medium, 10% heat-inactivated fetal bovine serum, 5% penicillin–streptomycin, 2 mM GlutaMAX, 10 mM HEPES, 1 mM sodium pyruvate, 0.1 mM non-essential amino acids, and 50 µM β-mercaptoethanol. The purity of BMMC was >80% (Supplementary Figure S1).

### 2.5. Cell Viability

A total of 1 × 10^5^ cells/100 µL/well of BMMCs were seeded in a 96-well plate and incubated at 37 °C for 48 h. BMMCs were cocultured with different concentrations of 1,3-butadiene and toluene for 24 h. A total of 10 µL CCK-8 reagent (Abcam, Cambridge, UK) was added in the culture plate and incubated at 37 °C for 4 h, followed by measuring the absorbance at 450 nm using a microplate reader (Sunrise, Tecan, Switzerland).

### 2.6. The Effect of 1,3-Butadiene and Toluene on Mast Cell Degranulation

A total of 2 × 10^5^ cells/mL of BMMCs was seeded in a 24-well plate and incubated at 37 °C for 24 h. Different concentrations of 1,3-butadiene and toluene were added into the cell culture for 1 h at 37 °C. Cells were harvested, supernatants were removed, and 160 µL of compound 48/80 (Cayman, Ann Arbor, MI, USA) (100 µg/mL in Tyrode’s buffer) was added. Cells were then incubated at 37 °C for 30 min. After centrifugation, 50 uL of the supernatant was added to the 96-well plate and mixed with 50 µL P-NAG (2 mM in 0.1 M citrate buffer, PH 4.5). Cell pellet was mixed with 160 µL 0.1% Triton X-100 (in Tyrode’s buffer) and incubated for 15 min on ice. A total of 50 µL of the mixture was then added to the 96-well plate and mixed with 50 µL P-NAG (2 mM in 0.1 M citrate buffer, PH 4.5). After incubation of the 96-well plate at 37 °C for 1 h, we added 100 µL of stop buffer to each well. Absorbance at 450 nm was measured using a microplate reader (Sunrise, Tecan, Switzerland). The level of released β-hexosaminidase was acquired as the percentage release based on the following formula: (stimulated supernatants/(stimulated supernatants + pellet) × 100 − unstimulated supernatants/(unstimulated supernatants + pellet) × 100) [11].

### 2.7. Statistical Analyses 

Statistical analyses were performed using Stata software 15.0 (StataCorp, College Station, TX, USA). Visual inspection of quantile–quantile plots of all variables to be analyzed showed no normal distribution. Quantitative data are therefore presented as medians plus the interquartile range. To account for the differences in age and sex between AD patients and HCs, the relationship between AD and log-transformed urine levels of VOC metabolites were examined using a multivariate linear regression model adjusted for age and sex. Bootstrap confidence intervals of regression coefficients were constructed using 1000 replications [12]. Correlations between serum IgE and the SCORAD index, and creatinine-corrected urine levels of VOC metabolites in AD patients, were performed with the non-parametric Spearman’s correlation analysis in 1000 bootstrap samples. All bootstrap confidence intervals were obtained through normal approximation. After Bonferroni’s correction for multiple comparisons as regards to 6 VOC metabolites, two-tailed *p* values < 0.008 were regarded as statistically significant. The comparison between in vitro experiments was undertaken using one-way analysis of variance (ANOVA).

## 3. Results

### 3.1. Baseline Characteristics of Adult AD Patients and HCs

Table 1 shows the baseline characteristics of adult AD patients and HCs. The median age of adult AD patients was higher than that of the controls (36 vs. 24 years). For AD patients, the median values of serum total IgE were 610 IU/mL, and for the SCORAD index, 30.2. Based on the SCORAD index, 13 (42%) patients were in the mild, 8 (26%) patients were in the moderate, and 10 (32%) patients were in the severe category in terms of disease severity. We started patient recruitment in October 2016. At that time, biologic therapies and JAK inhibitors were not approved for the treatment of AD. Therefore, there was no patient receiving such therapy in this study.

### 3.2. Urine Levels of VOC Metabolites in Adult AD Patients and HCs

**Figure 1:**
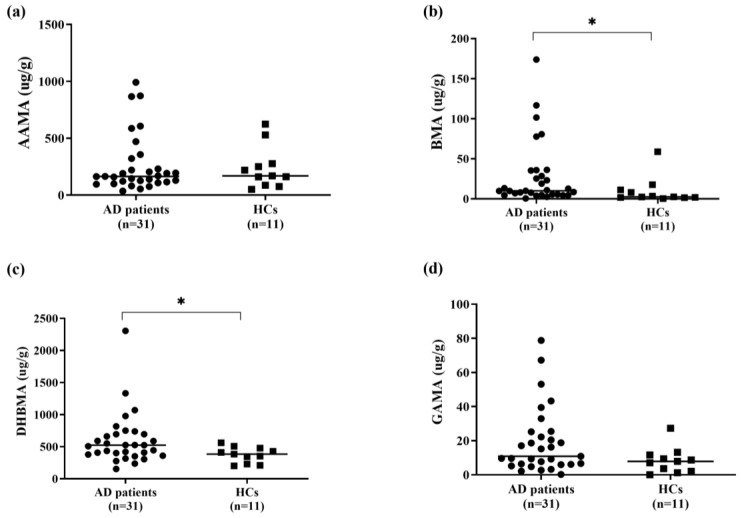
Creatinine-corrected urine levels of (**a**) AAMA, (**b**) BMA, (**c**) DHBMA, and (**d**) GAMA in adult AD patients (*n* = 31) and healthy controls (*n* = 11). * *p* < 0.05. AAMA, N-Acetyl-S-(2-carbamoylethyl)-L-cysteine; AD, atopic dermatitis; BMA, N-Acetyl-S-(benzyl)-L-cysteine; DHBMA, N-Acetyl-S-(3,4-dihydroxybutyl)-L-cysteine; GAMA, N-Acetyl-S-(2-carbamoyl-2-hydroxyethyl)-L-cysteine.

For AD patients, their creatinine-corrected urine levels of BMA and DHBMA appeared elevated compared with HCs (Figure 1).

### 3.3. Multivariate Linear Regression

Table 2 shows the regression coefficients of AD diagnosis in a multivariate linear regression adjusted for age and sex. The urine levels of BMA and DHBMA were higher in AD patients. But the differences were not statistically significant after correction for multiple comparisons.

### 3.4. Correlational Analyses

**Figure 2:**
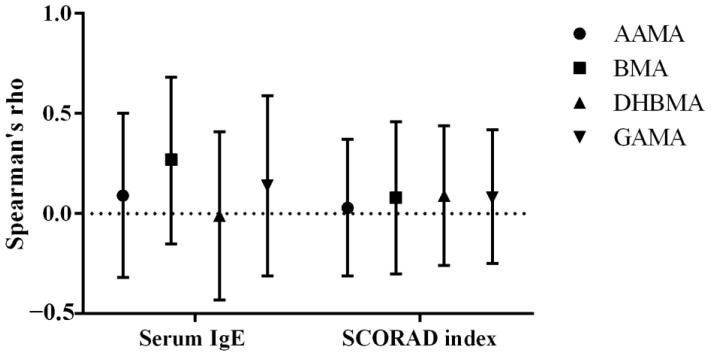
Spearman’s correlation coefficients and their 95% confidence intervals between serum IgE and SCORAD index and urine levels of creatinine-corrected VOC metabolites in AD patients. AAMA, N-Acetyl-S-(2-carbamoylethyl)-L-cysteine; AD, atopic dermatitis; BMA, N-Acetyl-S-(benzyl)-L-cysteine; DHBMA, N-Acetyl-S-(3,4-dihydroxybutyl)- L-cysteine; GAMA, N-Acetyl-S-(2-carbamoyl-2-hydroxyethyl)-L-cysteine; VOC, volatile organic compound.

As illustrated in Figure 2, BMA levels in urine were weakly associated with serum IgE levels. 

### 3.5. 1,3-Butadiene and Toluene Could Stimulate Mast Cell Degranulation

**Figure 3:**
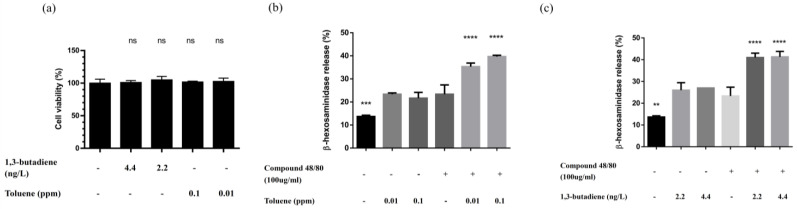
Effects of 1,3-butadiene and toluene on mouse bone marrow-derived mast cells (BMMCs): (**a**) Cell viability measured by CCK-8 assay after 24 h of treatment with 1,3-butadiene and toluene. Data are presented in percentages of the control. (**b**) The effect of toluene on BMMCs degranulation. β-hexosaminidase was measured in cell supernatants and lysates. The percentage of β-hexosaminidase release is shown. (**c**) The effect of 1,3-butadiene on BMMCs degranulation. β-hexosaminidase was measured in cell supernatants and lysates. The percentage of β-hexosaminidase release is shown. Values represent the means ± SEM from three independent experiments. ns, non-significant versus the control treated with the medium only. ** *p* < 0.01; *** *p* < 0.001; **** *p* < 0.0001 versus the group treated with compound 48/80 only. Statistical significance was determined by one-way ANOVA followed by Tukey’s multiple comparison test.

As illustrated in Figure 3a, 1,3-butadiene and toluene did not produce cytotoxicity on mouse BMMCs. 1,3-butadiene and toluene could stimulate the release of β-hexosaminidase in BMMCs as much as compound 48/80, a potent mast cell activator (Figure 3b,c) [13]. In addition, these VOCs could enhance the stimulatory effect of compound 48/80 on BMMCs.

## 4. Discussion

In this study on the possible association between VOC exposure and AD, we observed higher urine levels of BMA and DHBMA in AD patients compared with HCs. VOC exposure might contribute to the pathogenesis of AD in adults.

Our adult AD patients were younger than the general AD patient population (36 vs. 44 years) and with more males, which is somewhat different from previous studies [14]. Urine levels of both BMA, a toluene metabolite, and DHBMA, a 1,3-butadiene metabolite, appeared higher in our AD patients when compared with HCs. The associations remained after adjusting for age and sex. However, the results did not reach a statistically significant level after Bonferroni’s correction, and the correlations of these urine metabolites with disease activity were very weak. In terms of potential occupational exposure to VOCs, on the other hand, only two (0.6%) of the patients were at risk (one chemical plant worker and one retail worker).

The epidemiological relationship between VOC exposure and AD is less studied compared to asthma and has been explored mostly in children [1]. Herbarth et al. found that indoor renovation activities before birth and in early life were associated with the development of childhood eczema [15]. In 22 children with AD who had been followed up for 18 months, a 1 ppb increase in ambient total VOC concentration was associated with a 25.86% increase in AD symptoms on the following day [16]. In a double-blinded cross-over study on adult AD patients, controlled exposure to VOC mixtures led to increased transepidermal water loss and enhanced skin allergic reaction to house dust mites [17]. Our results are consistent with these findings, in that three of four detectable urine VOC metabolites in adult AD patients were numerically higher than those in HCs. However, the use of fossil fuels in the household, accompanied by an increased production of VOCs and NO2, was not associated with eczema when compared with the use of biomass in a study on 116 Bangladeshi children [18]. 

In terms of specific VOCs, toluene exposure has been linked to allergy and AD in several studies. Toluene treatment decreased filaggrin expression in human keratinocytes and a skin-equivalent model [19]. Our experiments also found that toluene could stimulate mast cells to degranulate. Repeated inhalation of toluene increased blood levels of IgE [20] and splenic expression of interleukin (IL)-4 and IL-12 [21] in mice which were immunized and then challenged with ovalbumin, but in these experiments, concentrations higher than those typical of households were used [22,23]. Topical high-concentration toluene application also marginally evoked ear inflammation and swelling in mice [24]. Kim et al. studied 30 AD children who had moved to a new building and their symptoms were elevated by 12.7% with a 1 ppb increment in ambient toluene concentrations [25]. In the Leipzig Allergy Risk Children Study on 200 children, higher total IgE levels in blood were associated with exposure to toluene (adjusted odds ratio: 3.3) [26]. Interestingly, toluene urine metabolite BMA levels were weakly correlated with serum total IgE levels in our AD patients. On the contrary, a study conducted in Malaysia measured ambient toluene concentrations at eight schools and found no association with dermal symptoms in students [27]. Taken together, the above results and our finding imply a potential contribution of toluene exposure to AD development in both children and adults. We also found that exposure to ambient 1,3-butadiene, which is emitted from both indoor and outdoor sources, was associated with adult AD. Its carcinogenicity through DNA adduct formation draws much attention in the literature, whereas its role in allergy pathogenesis has not been fully investigated. Only one time-series study of 22 asthmatic children found a positive association between ambient 1,3-butadiene exposure and asthma symptoms, although statistical significance was not reached [28]. Nevertheless, our experiments showed a stimulatory effect of 1,3-butadiene on mouse mast cells. More epidemiological and animal studies are needed to support the observations.

There are limitations to the present study. First, the small sample size is insufficient to detect small differences between AD patients and HCs with respect to multiple urine VOC metabolites. Nevertheless, HCs were difficult to recruit due to the fact that their selection criteria required no allergic diseases and no allergy to major allergens. Our results sill provide an insight into this critical issue. Second, some of the AD patients were not newly diagnosed and concurrent medications might have influenced the results. Finally, a causal relationship cannot be determined solely based on this cross-sectional study.

## 5. Conclusions

In conclusion, our results showed that the urine metabolites of some VOCs, such as 1,3-butadiene and toluene, were elevated in adult AD patients. 1,3-butadiene and toluene could stimulate mast cells to degranulate. This suggested a potential impact of VOC exposure on adult AD pathogenesis. Avoidance of VOC exposure should be considered in AD patients.

## Figures and Tables

**Table 1:** Baseline characteristics of the study participants.

	AD Patients (*n* = 31)	HCs (*n* = 11)
Age, median (IQR)	36 (28, 43)	24 (20, 34)
Female	13 (42%)	7 (64%)
Allergic rhinitis	14 (45%)	0 (0%)
Asthma	8 (26%)	0 (0%)
Smoking	7 (23%)	2 (18%)
Serum total IgE (IU/mL)	610 (181, 5000)	NA
SCORAD index	30.2 (12.8, 52.9)	NA
Systemic medications		
Antihistamine	28 (90%)	NA
Oral corticosteroids	16 (52%)	NA
Azathioprine	4 (13%)	NA
Cyclosporine	3 (10%)	NA
Methotrexate	10 (32%)	NA
Omalizumab	3 (10%)	NA

AD, atopic dermatitis; HC, healthy control; NA, not available; IQR, interquartile range.

**Table 2:** The relationships between adult AD and log-transformed urine levels of VOC metabolites ^†^ in a multivariate linear regression model after adjustment for age and sex.

Coefficients (95% CI ^‡^)	AAMA	BMA	DHBMA	GAMA
Age	0.007 (−0.022, 0.035)	0.014 (−0.030, 0.059)	0.002 (−0.016, 0.021)	0.015 (−0.023, 0.053)
Male sex	0.037 (−0.508, 0.583)	−0.118 (−0.942, 0.705)	−0.017 (−0.339, 0.306)	−0.095 (−0.925, 0.736)
AD patients ^§^	−0.035 (−0.721, 0.651)	1.100 (0.041, 2.158)	0.364 (0.003, 0.725)	0.900 (−0.175, 1.974)

AAMA, N-Acetyl-S-(2-carbamoylethyl)-L-cysteine; AD, atopic dermatitis; BMA, N-Acetyl-S-(benzyl)-L-cysteine; CI, confidence interval; DHBMA, N-Acetyl-S- (3,4-dihydroxybutyl)-L-cysteine; GAMA, N-Acetyl-S-(2-carbamoyl-2-hydroxyethyl) -L-cysteine; VOC, volatile organic compounds. ^†^ Creatinine-corrected. ^‡^ Bootstrap confidence intervals constructed using 1000 replications. ^§^ Compared with the reference group, healthy controls.

## Data Availability

The data that support the findings of this study are available from the corresponding author.

## Supplementary Materials

The following supporting information can be downloaded at: https://www.mdpi.com/article/10.3390/biomedicines12071419/s1, Table S1: Limit-of-detection (LOD) values for urine levels of VOC metabolites determined by liquid chromatography–mass spectrometry. Figure S1. Examples of bone marrow-derived mast cells (BMMCs) stained with PE-anti-c-kit and FITC-anti-FcεRI analyzed by flow cytometry. Numerically, >80% of cells are mast cells (CD117^+^FcεRIa^+^ cells).
