# Supplementary material for: Exposure to Volatile Organic Compounds May Contribute to Atopic Dermatitis in Adults
Source: Biomedicines. 2024 Jun 26;12(7):1419. doi: 10.3390/biomedicines12071419 (PMC11274632; doi:10.3390/biomedicines12071419)
Supplement: Supplementary file 1 [file biomedicines-12-01419-s001.zip › biomedicines-3032264-supplementary.pdf]

Supplementary materials

Table S1. The limit of detection (LOD) values for urine levels of VOC metabolites determined by liquid chromatography–mass spectrometry.

VOC metabolites	LOD value (ppb)
2,4-DPMA	0.1
AAMA	0.2
BMA	0.01
DHBMA	0.05
GAMA	0.05
PMA	0.05

2,4-DPMA, N-Acetyl-S-(2,4-dimethylphenyl)-L-cysteine; AAMA, N-Acetyl-S-(2-carbamoylethyl)-L-cysteine; BMA, N-Acetyl-S-(benzyl)-L-cysteine; DHBMA, N-Acetyl-S- (3,4-dihydroxybutyl)-L-cysteine; GAMA, N-Acetyl-S-(2-carbamoyl-2-hydroxyethyl)-L-cysteine; PMA, N-Acetyl-S-(phenyl)- L-cysteine; VOC, volatile organic compounds.

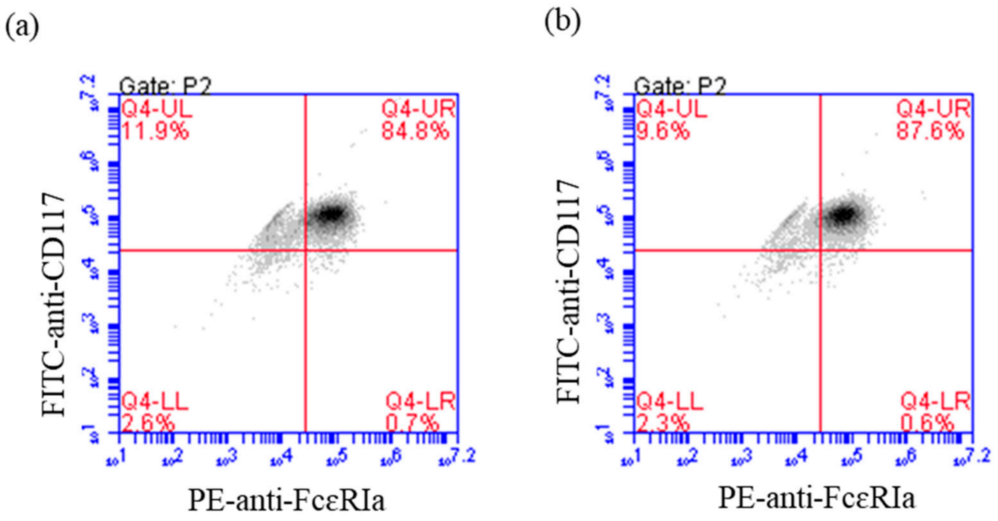

Figure S1. Examples of bone marrow-derived mast cells (BMMC) stained with PE-anti-c-kit and FITC-anti-FcεRI analyzed by flow cytometry. Numerically, > 80% of cells are mast cells (CD117$^+$FcεRIa$^+$ cells.
